# Comparison of the Potential Abilities of Three Spectroscopy Methods: Near-Infrared, Mid-Infrared, and Molecular Fluorescence, to Predict Carotenoid, Vitamin and Fatty Acid Contents in Cow Milk

**DOI:** 10.3390/foods9050592

**Published:** 2020-05-06

**Authors:** Julien Soulat, Donato Andueza, Benoît Graulet, Christiane L. Girard, Cyril Labonne, Abderrahmane Aït-Kaddour, Bruno Martin, Anne Ferlay

**Affiliations:** 1 Université Clermont Auvergne, INRAE, VetAgro Sup, UMR Herbivores, F-63122 Saint-Genès-Champanelle, France; julien.soulat@inrae.fr (J.S.); donato.andueza@inrae.fr (D.A.); benoit.graulet@inrae.fr (B.G.); cyril.labonne@inrae.fr (C.L.); bruno.martin@inrae.fr (B.M.); 2 Agriculture and Agri-Food Canada, Sherbrooke Research and Development Centre, Sherbrooke, QC J1M 0C8, Canada; Christiane.Girard@agr.gc.ca; 3 Université Clermont Auvergne, INRAE, VetAgro Sup, UMRF, 15000 Aurillac, France; abderrahmane.aitkaddour@vetagro-sup.fr

**Keywords:** carotenoids, fatty acids, vitamins, milk, infrared, front face fluorescence, synchronous fluorescence

## Abstract

The objective of this work is to compare the ability of three spectroscopy techniques: molecular fluorescence, near-infrared (NIR), and mid-infrared with attenuated total reflectance (MIR-ATR) spectroscopy to predict the concentrations of 8 carotenoids, 6 vitamins and 22 fatty acids (FA) in cow’s milk. A dataset was built through the analysis of 242 frozen milk samples from different experiments. The milk compounds were analysed using reference methods and by NIR, MIR-ATR, and fluorescence to establish different predictive models. NIR spectroscopy allowed for better prediction of cis9-β-carotene, β-cryptoxanthin and the sum of carotenoids than the other techniques, with a coefficient of cross-validation in calibration (R^2^CV) > 0.60 and a coefficient of determination in validation (R^2^V) > 0.50. Their standard errors of prediction (SEP) were equal to 0.01, except for the sum of carotenoids (SEP = 0.15). However, MIR-ATR and fluorescence seem usable for the prediction of lutein and all-trans-β-carotene, respectively. These three spectroscopy methods did not allow us to predict (R^2^CV < 0.30) vitamin contents except, for vitamin A (the best R²CV = 0.65 with NIR and SEP = 0.15) and α-tocopherol (the best R²CV = 0.56 with MIR-ATR and SEP = 0.41), but all R²V were <0.30. NIR spectroscopy yielded the best prediction of the selected milk FA.

## 1. Introduction

Dairy products are a good source of nutrients (proteins and fatty acids (FA)), and micronutrients (minerals, vitamins, and carotenoids). They are thus of great nutritional interest for human health [1,2]. Carotenoids, usually present in milk in six molecular forms (13cis-, 9cis-, and all-trans-β-carotenes, and lutein, zeaxanthin and β-cryptoxanthin), are involved in the nutritional and sensory qualities of dairy products [3]. Some of them (β-carotenes and β-cryptoxanthin) are also vitamin A precursors and protect the other milk compounds, especially lipids, from oxidation [4]. Among the different milk macronutrients, fat plays also a major role in the nutritional and sensory qualities of dairy products [5,6]. Moreover, the different FA present in milk fat can have either a positive or a negative role on human health [2]. When consumed in excess, C16:0 is linked to a risk of hypercholesterolemia and coronary heart disease [7]. However, milk fat also contains small amounts of polyunsaturated FA (PUFA), such as conjugated linoleic acids (CLA), and n-3 FA, which can have positive effects on human health [5]. Milk micronutrient (carotenoids and vitamins) content and FA composition vary according to feeding systems [8,9]. Indeed, milk from grazing cows is naturally richer in carotenoids, vitamins A and E, cis9-C18:1, trans11-C18:1, cis9trans11-C18:2, and C18:3 n-3 and poorer in C16:0 than milk from cows fed with preserved forages (e.g., hay, silage) [8,10].

Considering the growing need for information by consumers on the nutritional quality of foods, it is important for the dairy sector (producers and industries) to characterise the nutritional composition of cow’s milk. The reference chemical methods, often based on chromatographic methods, for measuring carotenoids, vitamins, and FA composition, are the most accurate methods because they can effectively measure the individual content of each milk compound. However, these methods are generally time-consuming and require the use of pollutant solvents. In case of routine and extensive use, spectroscopy techniques (near and mid-infrared (NIR and MIR) and molecular fluorescence) could be an alternative to these chemical methods, even if they only predict the contents. These three analytical techniques are non-invasive, rapid, non-destructive, non-contaminating, cheap to implement, and could provide a multiparametric determination from one sole analysis of a given sample. These techniques also allow us to reduce the human resources required for the analyses. However, we must build large databases comparing the concentrations of the compounds of interest obtained by chemical analyses with the spectra obtained by spectroscopy analyses of the same milk samples to establish prediction models. Milk FA have already been successfully predicted using the NIR [11] and the MIR spectroscopy techniques [12,13,14]. Concentrations of β-carotene, the sum of carotenoids, vitamins A and E in cheeses [15], and vitamins A and E in ewe’s milk [16] have been predicted using NIR spectroscopy. To date, the milk concentrations of carotenoids and vitamins have not been predicted via MIR spectroscopy, which is among the most preferred methods for milk and dairy product analysis [17]. Milk recording organizations routinely use MIR to predict milk fat, protein, lactose, and urea content for their subscribers. There are different methods used for MIR spectroscopy, including attenuated total reflectance (ATR) [18]. ATR allows one to limit the absorption by water, thus limiting the penetration of infrared radiation into the sample [18,19]. However, the performance of MIR-ATR in predicting some milk compounds has not been extensively reported in the literature [18,19].

Molecular fluorescence spectroscopy has been used, for example, to discriminate ewe’s milk samples from different feeding systems [20] or to quantify the riboflavin in yogurt [21]. Nevertheless, few studies have used molecular fluorescence spectroscopy to quantify nutritional compound concentrations in milk and dairy products [21,22,23] although this technology could be adapted for predicting the compounds present in small concentrations. According to Sádecká and Tóthová [24], this technique is 100 to 10,000 times more sensitive than infrared spectroscopy (NIR and MIR). Meanwhile, no studies have compared these three spectroscopy techniques to predict different milk compound concentrations from the same milk samples.

According to the spectroscopy technique used, the nature of the milk samples (liquid, oven-dried, fresh, thawed, or homogenized) could have an effect on the spectra [12,19,25], particularly when freezing and thawing samples. However, according to Coppa et al. [12], the quality of FA prediction for liquid samples was not impacted when thawed samples were used with the MIR method, whereas the quality of prediction was significantly lower with the NIR method. Otherwise, Aernouts et al. [19] reported instead that when all samples were similarly conditioned, the MIR performance was not impacted. Furthermore, these authors showed that the homogenization of thawed samples could minimize the effect of freezing samples on MIR performance.

Thus, the aim of this study was to compare the ability of NIR, MIR-ATR, and molecular fluorescence techniques to predict and quantify compounds of nutritional interest (carotenoids, vitamins, and FA) in cow’s milk from thawed samples.

## 2. Materials and Methods

### 2.1. Experiments and Cow Diets

One hundred and eighty individual cow’s milk samples and 62 bulk milk samples were selected for the four experiments described in Appendix A. These samples were previously analysed for their FA, carotenoid, and vitamin composition using the reference chemical methods (Table 1). Each milk sample, from these four experiments, was stored at −20 °C, without a bronopol preservative before the analyses.

### 2.2. Chemical and Spectroscopy Analyses

#### 2.2.1. Reference Chemical Analyses

The milk samples were analysed to quantify their carotenoids, vitamins and FA composition. The milk contents of 13cis-β-carotene, 9cis-β-carotene, all-trans-β-carotene, β-cryptoxanthin, lutein, zeaxanthin, retinol (vitamin A), and α- and γ-tocopherol (vitamins E) were simultaneously measured at 325, 292, and 450 nm using an Ultra-Performance Liquid Chromatography equipped with a 150 × 2.1 mm HSS T3, 1.8-µm column and a photo-diode array detector (Affinity system, Waters Corp., Saint-Quentin-en-Yvelines; France) [3]. Milk vitamin B_9_ (folates) and vitamin B_12_ (cobalamins) were measured by a radioassay (SimulTRAC^®^ B12/FOLATE-S, MP Biomedicals, Solon, OH, USA) according to Duplessis et al. [26]. For the FA analysis, the milk samples were first lyophilized, and then the FA were methylated according to the method of Ferlay et al. [27]. The FA methyl esters were injected into a Trace-GC 2000 series gas chromatograph equipped with a flame ionisation detector (Thermo Finnigan, Les Ulis, France). The FA methyl esters from all milk samples were separated on a 100 m × 0.25-mm i.d. fused-silica capillary column (CP-Sil 88, Chrompack, Middelburg, the Netherlands). In our study for each milk sample, the FA concentrations were expressed as the percentage of the total FA or as g/kg of milk.

The standard errors of laboratory (SEL) for the carotenoids and vitamins were estimated according to the methodology proposed by AOAC [28], and the SEL values for the FA were taken from Coppa et al. [25].

#### 2.2.2. Spectroscopic Analyses

Milk samples were thawed in a bath at 30 °C and shaken slowly to homogenize them by hand; the samples handled away from direct light to limit the degradation of carotenoids and vitamins. Then, they were divided into three sub-samples before spectral analyses via NIR, MIR, and fluorescence spectroscopy and were analysed the same day. In our study, 242 milk samples were analysed by NIR and MIR spectroscopy techniques. Only 229 milk samples were analysed by fluorescence spectroscopy since for 13 milk samples, the remaining volume was too low to perform this analysis.

1. Near-Infrared Spectroscopy Analysis

After homogenization, each thawed milk sample (0.5 mL) was oven-dried at 30 °C for 24 h on a glass microfiber filter (Whatman GF/A, 55 mm, Cat.No.1820 055, Whatman International Ltd., Maidstone, UK) and placed in a 50-mm-diameter ring cup (DESIR method) according to Thyholt and Isaksson [29]. The precision of the DESIR method for predicting milk FA was higher than that obtained when the liquid samples were used [25]. The filters were scanned in reflectance mode at 2 nm intervals from 400 to 2500 nm using a FOSS NIRSystems model 6500 NIR scanning spectrometer (FOSS NIRSystems, Silver Spring, MD, USA) equipped with an autocup module and controlled via the ISIscan software version 2.21 (Infrasoft International LLC, Stage College, PA, USA). Each NIR spectrum was time-averaged from 32 scans and compared with the average of 32 scan measurements of a ceramic reference.

2. Mid-Infrared Spectroscopy Analysis

Milk samples were analysed using a Varian 3100 FT-IR Excalibur Series Fourier-transform spectrometer (Varian Inc., Palo Alto, CA, USA). The milk samples were shaken to homogenize them by hand. Then, the samples (3 mL) were poured into water heated Trough Attenuated Total Reflectance (ATR) Top Plate (Gateway™ ATR Water Heated Trough Top Plate). This ATR cell had a reflection horizontal crystal made of Zinc Selenide (ZnSe) with a number of reflections of six, an incidence angle of 45°, and a thermocirculated water system. To improve the signal-to-noise ratio, 16 scans were averaged for each spectrum. The milk MIR-ATR spectra were recorded at 20 °C between 4000 to 525 cm^−1^ (2500 to 19,048 nm) with a resolution of 2 cm^−1^. Before each analysis, blanks with the empty ATR cell were scanned. Finally, to consider the cleaning effects on the crystal ATR cell, the blank spectra were removed to the corresponding milk sample spectra. Between each measurement, the ATR cell was thoroughly washed with ethanol and demineralized water and then dried with compressed air (Atlas Copco, Nacka, Sweden).

3. Molecular Fluorescence Spectroscopy Analysis

Molecular fluorescence spectra were recorded using a Fluoromax^®^-4 (Jobin-Yvon, Longjumeau, France) mounted with an angle front-surface accessory composed of two mirrors. After homogenization, milk samples (2.5 mL) were placed in quartz tubes (3 × 1 × 1 cm). For the prediction of milk carotenoids, vitamin and FA concentrations, two methods were applied: front-face synchronous fluorescence and classical front-surface fluorescence using the excitation wavelengths and the emission spectra of vitamin A, tryptophan, vitamin B_2_, carotene and lycopene, chlorophyll and fluorescent oxidation products (FOP) [20,24,30,31]. The samples (three replicates) in quartz tubes were shaken between each replication acquisition. Finally, each sample featured three fluorescence spectra per method.

### 2.3. Calculations and Statistical Analyses

The calibrations were calculated using the WiniISI III version 1.60 software (Infrasoft International, South Atherton St. State College, PA, USA) for the three spectroscopy techniques (NIR, MIR-ATR, and fluorescence) used. The milk samples were randomly divided into calibration and validation sets (Table 2 and Table A1). The same calibration and validation sets were used for the three spectroscopy techniques.

**Figure A1:**
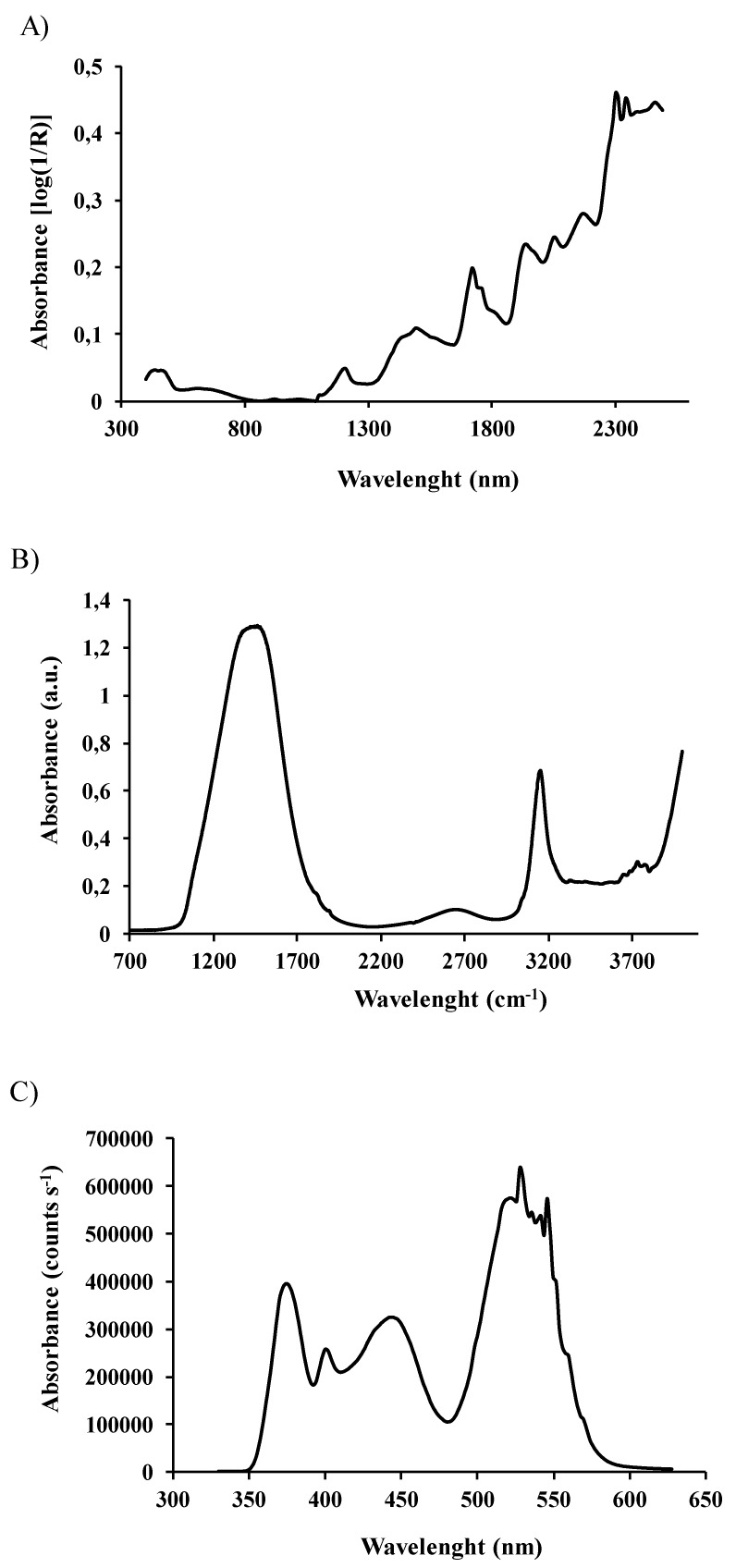
Illustration of each average raw spectrum obtained by near-infrared (**A**), mid-infrared using attenuated total reflectance cell (**B**), and synchronous fluorescence method (**C**). (R: reflectance).

To establish the calibration models for each milk compound studied, the part of the spectra used varied between:

- NIR spectroscopy (Figure A1A): 400 to 2500 nm and 700 to 2500 nm;
- MIR-ATR spectroscopy (Figure A1B): 4000 to 700 cm^−1^ (i.e., 2500 to 14,286 nm);
- Front face fluorescence spectroscopy: 470 to 750 nm for carotene, 400 to 600 nm for chlorophyll, 370 to 600 nm for FOP, 490 to 750 nm for lycopene, 340 to 450 nm for tryptophan, 340 to 600 nm for vitamin A, and 400 to 730 nm for vitamin B_2_. The excitation wavelengths were carotene = 450 nm; chlorophyll = 365 nm; FOP = 350 nm; lycopene = 473 nm, tryptophan = 290 nm, vitamin A = 320 nm; and vitamin B_2_ = 380 nm;
- Synchronous fluorescence spectroscopy (Figure A1C): 330 to 630 nm. The wavelengths of excitation were between 250 and 550 nm.

The modified partial least squares (MPLS) regression method was used to obtain all calibration equations for the three spectroscopy techniques and all compounds studied in the milk. According to Shenk and Westerhaus [32], the MPLS regression method is more stable and accurate than the standard partial least squares regression method. To optimize the calibration models, different smoothing and derivative spectral treatments were employed along with scattering correction methods. For each calibration model established from the different spectral processing and scattering corrections, the coefficient of determination in calibration (R^2^C) and the standard error of calibration (SEC) were calculated. Moreover, a cross validation of the prediction model was performed using the calibration dataset. The calibration set was first randomized and then divided into four groups. Each group was temporarily removed and predicted using calibration developed from the other samples. The statistics associated with the model were then calculated.

The calibration equation was selected for each constituent on the basis of the highest coefficient of determination in cross validation (R^2^CV) and the lowest standard error of cross validation (SECV). Samples were considered outliers (T) if the residual value between the reference method and the predicted value was larger than 2.5 times the standard error of calibration [33]. As described by Andueza et al. [33], spectra were considered outliers if the standardized Mahalanobis distance (H) between the sample and the average spectrum was higher than three. Two passes of outlier elimination were allowed. On completion of calibration, the model was applied to the validation set. The coefficient of determination in external validation (R^2^V) and the standard error of prediction (SEP) were calculated. The standard error of prediction for each model was then separated into the bias and the SEP corrected for bias (SEPc). The significance of the bias for each model was tested if the bias was different from 0 according to van Reeuwijk and Houba [34]. The SEPc differences between the three spectroscopy techniques were tested according to Fearn [35].

Pearson’s correlations were performed between the different milk compounds in the calibration set.

## 3. Results

The samples used in the calibration and validation sets are described in Table 2 and Table S1. The mean and standard deviation values for each milk compound were similar for both data sets (calibration and validation). For each spectroscopy technique, only the best calibration equation (highest R^2^CV and R^2^V) for each milk compound was presented, as well as its scattering correction and spectral processing (Table 3, Table 4 and Table 5 and Table A2). For the NIR spectroscopy, the highest R^2^CV and R^2^V values were obtained using the spectra with, a range between 400 and 2500 nm (Figure A1A). The calculation of bias and SEPc allowed us to compare the three spectrometry techniques (Table 6 and Table A3).

### 3.1. Prediction of Carotenoids in Milk

The prediction of carotenoids was generally better with NIR spectroscopy providing the best prediction values for cis9-β-carotene, β-cryptoxanthin and zeaxanthin, R^2^CV > 0.70, and R^2^V > 0.60 (Table 3). All-trans-β-carotene, lutein and the sum of carotenoids were R^2^CV ≥ 0.60, but their R^2^V values were below 0.35, except for the sum of carotenoids (R^2^V = 0.50). The SEPc values of cis9-β-carotene and β-cryptoxanthin were lower (*p* < 0.05) with NIR spectroscopy than molecular fluorescence spectroscopy, but there was not a significant difference (*p* > 0.05) between the NIR and MIR-ATR spectroscopy techniques. The bias values for both these carotenoids were significantly different from zero only under NIR spectroscopy (Table 6). For lutein, the MIR-ATR spectroscopy had a similar R^2^CV to NIR but a higher R^2^V (Table 4). The SEPc obtained by MIR-ATR spectroscopy for this carotenoid was significantly lower (*p* < 0.05) than that with NIR spectroscopy but not significantly different than the one obtained via molecular fluorescence spectroscopy (Table 6).

The best prediction values for cis13-β-carotene and the sum of β-carotenes were obtained with fluorescence spectroscopy (Table 3). According to our results, the model of cis13-β-carotene was characterised by low R^2^CV and R^2^V values (lower than 0.35), regardless of the spectroscopy technique. All carotenoid bias values obtained by molecular fluorescence were significantly different from zero.

Fluorescence spectroscopy also allowed a better prediction of all-trans-β-carotene with the highest R^2^V (Table 3). The SEPc value of this carotenoid was significantly lower (*p* < 0.05) with fluorescence spectroscopy than with MIR-ATR spectroscopy.

### 3.2. Prediction of Vitamins in Milk

The prediction quality of the vitamins in milk was relatively low regardless of the spectroscopy technique used. In this study, except for α-tocopherol and vitamin A, the predicted vitamins had an R^2^CV and R^2^V below 0.30. According to our results, NIR spectroscopy offered the best prediction of vitamin A (R^2^CV = 0.65, Table 4). The bias value for this vitamin was significantly different from zero when only using the molecular fluorescence (Table 6).

MIR-ATR spectroscopy provided the best predictions for α-tocopherol, with R^2^CV = 0.56 and R^2^V = 0.40 (Table 4). The SEPc was significantly lower (*p* < 0.05) under MIR-ATR spectroscopy compared to NIR spectroscopy and not significantly different (*p* > 0.05) when compared to fluorescence spectroscopy. The bias values for α-tocopherol were significantly different from zero for all three spectroscopy techniques (Table 6).

Our results show that the models that predict the concentrations of γ-tocopherol, the sum of tocopherols, and both vitamins B (B_9_ and B_12_) are characterised by very low R^2^V and R^2^CV, whatever the spectroscopy technique used (Table 4).

### 3.3. Prediction of Fatty Acids in Milk

In our study, the statistical values of FA prediction were generally better using NIR spectroscopy, regardless of the unit used. Independently of the unit, C6:0, C8:0, C10:0, C12:0, C14:0, C16:0, trans11-C18:1 and the sum of trans FA had an R^2^CV and R^2^V above 0.70 (Table 5 and Table A2). As shown by the NIR spectroscopy, the R^2^CV and R^2^V values were also above 0.70 for cis9-C18:1, sum of MUFA, and the sum of PUFA, when these FA were expressed as a percentage of the total FA (Table 5), as well as and for cis9trans11-C18:2 and the sum of SFA when expressed as g/kg of milk (Table A2). With NIR spectroscopy, the sum of SFA (expressed as percentage of the total FA) had an R^2^CV equal to 0.65 and an R^2^V ≥ 0.80 (Table 5). Independently of the FA unit, for C6:0, C8:0, C10:0, C12:0, C14:0, C16:0, cis9-C18:1, trans11-C18:1 as well as cis9trans11-C18:2, the sum of SFA, MUFA, PUFA and trans FA, the SEPc values obtained with NIR spectroscopy were significantly lower (*p* < 0.05) than those obtained with the two other techniques (Table 6 and Table A3). The bias values for these FA were not significantly different from zero, except for the sum of SFA and MUFA when these FA were expressed as g/100 g.

Independently of the unit, the FA predictions obtained with MIR-ATR spectroscopy were poor, with an R^2^V below 0.30 (Table 5 and Table A2). Fluorescence spectroscopy allowed a better prediction of C18:2 n-6, C18:3 n-3, cis9trans11-C18:2, n-3 FA, and n-6 FA than other spectroscopy techniques, according to the R^2^CV, when the FA were expressed as a percentage of the total FA (Table 5). However, with this technique, the R^2^V values were very low (between 0.02 and 0.50). The R^2^CV of the C18:3 n-3 prediction was higher for the molecular fluorescence than the other techniques, when this FA was expressed as g/kg of milk, but the R^2^V was also very low (Table A2).

Whatever the FA unit and the spectroscopy technique used, our results show that the predictions of C4:0, C18:0, trans10-C18:1, trans11cis15-C18:2, C18:2 n-6, C18:3 n-3, n-3 FA, and n-6 FA are characterised by an R^2^CV and R^2^V below 0.60 (Table 5 and Table A2). Regardless of the FA unit used, the prediction of cis9trans11-C18:2 was also low (R^2^CV and R^2^V below 0.60), however, the R^2^CV obtained, when this FA was expressed as g/kg of milk was equal to 0.67 (Table A2).

### 3.4. Correlations among Milk Compounds

The cis9-β-carotene was highly correlated with β-cryptoxanthin, lutein and zeaxanthin (absolute value of Pearson’s correlations (|r|) ranging from 0.84 to 0.95, Table A4). Lutein and β-cryptoxanthin were also highly correlated with zeaxanthin and the sum of carotenoids (|r| ranging from 0.84 to 0.87). The sum of carotenoids was highly correlated with cis13-β-carotene (|r| > 0.81). A correlation above 0.91 was observed between α-tocopherol and γ-tocopherol and the sum of tocopherols. High correlations were found for different SFA such as C6:0, C8:0, C10:0, C12:0 and C16:0 (|r| ranging from 0.81 to 0.99). The SFA and MUFA were also highly correlated with C6:0, C8:0, C10:0, C12:0, C14:0 and C16:0 (|r| ranging from 0.88 to 0.94). Further, trans11-C18:1 and cis9trans11-C18:2 were highly correlated with PUFA (|r| > 0.90). In our study, the carotenoids and the vitamins were weakly correlated with the FA (|r| ranging from 0.002 to 0.58).

## 4. Discussion

### 4.1. Prediction of Carotenoids and Vitamins

To our knowledge, there are few studies that use spectroscopy techniques to predict carotenoids and vitamins in dairy products. This study is the first to attempt to predict the concentrations of different carotenoids and vitamins in cow’s milk via three spectroscopy techniques: NIR, MIR-ATR, and fluorescence. NIR spectroscopy was the sole technique used to predict vitamins and carotenoids in cheeses [15] and in ewe’s milk [16]. Lucas et al. [15] obtained a better prediction of the sum of β-carotenes in cheeses with an R^2^CV and R^2^V above 0.92 for fresh cheese than our results with cow’s milk (R^2^CV and R^2^V equal to 0.51 and 0.20, respectively). However, the variability (coefficient of variation, CV = 87.5%) of the database of cheeses reported by Lucas et al. [15] was higher than that of the database used in this study (CV = 25.0%). Moreover, the concentration of β-carotene in milk (0.29 μg/mL) was low compared to that of fresh cheese (0.80 mg/kg). The low concentration of this carotenoid in milk, as well as the lower variability among samples in the database could explain the low quality of our predictions compared to those obtained from cheeses [15]. With NIR spectroscopy, the best predictions of β-carotene concentration were obtained using other matrices with R^2^CV values above or equal to 0.80, such as in Chinese kale [36]. To our knowledge, the other carotenoids considered in our study have never been predicted in milk samples using MIR-ATR and fluorescence spectroscopy. However, different studies showed that it is possible to predict concentrations of β-cryptoxanthin, lutein, zeaxanthin [36,37], the sum of carotenes [38] and the sum of carotenoids [37,39,40] in vegetables using NIR spectroscopy. The equations used to predict the concentrations of these different carotenoids in vegetable samples are more precise than those applied in the current study. As observed for β-carotene, the concentrations of these carotenoids are higher in vegetables than in milk. The low concentrations of these carotenoids in milk seemingly problematize their prediction via NIR spectroscopy. In our study, the SEPc values for the carotenoids were generally higher than the bias values. According to the SEPc values, which were similar for the prediction of cis13-β-carotene, the sum of β-carotenes, and zeaxanthin, it is not possible to privilege any one spectroscopy technique. According to our results, NIR spectroscopy can be used to predict the concentration of cis9-β-carotene, β-cryptoxanthin and the sum of carotenoids because it provides the lowest SEPc and the highest R^2^CV and R^2^V values, whereas the MIR-ATR and fluorescence techniques can be used to predict concentrations of lutein and all-trans-β-carotene.

With NIR spectroscopy, the R^2^CV of the equations for vitamin A and α-tocopherol were higher and more similar than those obtained by Revilla et al. [16] for ewe’s milk samples. However, for both vitamins, the R^2^V obtained by Revilla et al. [16] was higher than that in the current study. Using the MIR-ATR spectra, we obtained higher R^2^CV values for the α-tocopherol equation than Revilla et al. [16] with NIR spectroscopy, but the R^2^V was slightly lower. Our predictions of the milk vitamin A and α-tocopherol were better than the results of Lucas et al. [15] for cheese samples. The mean concentration of vitamin A was lower in cow’s milk than in ewe’s milk, while the opposite was true for the mean concentration of α-tocopherol [16]. The concentration of these vitamins was lower in milk than in cheese [15]. These different results show that the low concentrations of vitamin A or α-tocopherol are not the only factors modifying the precision of predictions. One explanation could be linked to the sample matrix. According to our results, it is not possible to privilege a single spectroscopy technique to predict the milk concentration of γ-tocopherol, the sum of tocopherols, or vitamin A. However, our results demonstrate that MIR-ATR spectroscopy provides the best prediction of the α-tocopherol concentration in milk. These results should be confirmed, however, as this study it is the first time that these vitamins in milk were predicted by MIR-ATR and fluorescence.

To our knowledge, this is also the first attempt to predict milk vitamin B_9_ and B_12_ concentrations using these spectroscopy techniques. Pires et al. [41] succeeded in predicting vitamins B_9_ and B_12_ (R^2^CV and R^2^V above 0.95) from premix animal food using NIR spectroscopy. However, in cow’s milk, the concentrations of these vitamins are very low compared to those measured in premix food by Pires et al. [41]. Moreover, the very limited number of milk samples in our study likely precluded the chance to obtain precise equations to predict concentrations for these two vitamins in milk using a spectroscopy technique.

Molecular fluorescence spectroscopy is considered more sensitive than infrared techniques to detect compounds of a low concentration [24]. However, we did not obtain better prediction equations for carotenoids and vitamins using this technique. Conversely, we obtained better results with NIR spectroscopy, although this last technique is known to poorly predict compounds with low concentrations. It is possible that the concentrations of the studied molecules were correlated with the other compounds present in milk. For example, the zeaxanthin was very correlated (r = 0.95) to the cis9-β-carotene, as well as the α-tocopherol to the sum of tocopherols (r = 0.97, Table A4). If such correlations exist, the concentrations of some of the studied molecules by infrared techniques could be indirectly predicted by association with these other compounds. According to our slightly |r| values, FA cannot predict carotenoids and vitamins indirectly (Table A4). It is also possible that some carotenoids or vitamins were predicted indirectly by other carotenoids or vitamins in greater concentration in milk. Another explanation for the results obtained via NIR spectroscopy is that, in our NIR models, we also considered the absorbance at visible wavelengths because the studied molecules absorbed in the visible wavelengths [42]. According to our observations (data not shown), the loadings of the visible segment are the most important for the carotenoids and vitamins. It is well known that the absorbance values of the visible segment are most sensitive that those in the near infrared segment [42]. This could partially explain the best predictions obtained for carotenoids and vitamins with NIR spectroscopy although these compounds are in very small quantity in milk. When the relative uncertainty was calculated according to De la Roza-Delgado et al. [43], obtained values were similar for the three spectroscopy methods for all carotenoids and vitamins (data not shown) showing that no differences between methods were observed for uncertainty values associated with potential interferences (specificity).

Moreover, the quality of predicting carotenoids and vitamins could also be improved by analysing fresh instead of frozen milk samples. Indeed, these molecules are very sensitive and can be rapidly degraded by oxidation [44]. Moreover, freezing could have altered the milk samples and thus their spectra for MIR-ATR and fluorescence techniques. In cheese, when using the DESIR method for NIR spectroscopy, the prediction quality of β-carotene, vitamin A and α-tocopherol is lower when the analyses are performed on in frozen rather than fresh samples [15].

### 4.2. Prediction of Fatty Acids

A prediction of cow’s milk FA has already been performed using NIR and MIR spectroscopy [12,13,14]. To our knowledge, molecular fluorescence spectroscopy has never been used to predict FA and no study has used the same dataset to predict FA via the spectra of different spectroscopy techniques. In our study, better predictions of FA were generally obtained using NIR spectroscopy, regardless of the FA unit used. However, the R^2^CV and R^2^V obtained in our study with NIR spectroscopy were generally lower than those obtained by Coppa et al. [12,25] in cow’s milk when the FA are expressed as the percentage of the total FA. These differences in prediction quality can be explained by the fact that, in these two studies, the variability of the datasets was higher. The dataset used to predict FA in our study had lower variability than the datasets used by Coppa et al. [25] and had similar variability than the datasets used by Soyeurt et al. [14].

The prediction of FA obtained in our study with MIR-ATR spectroscopy is very poor compared to that of many studies, whatever the FA unit used [14,45,46]. Generally, in those studies, the predictions of FA with MIR spectroscopy were better when using the unit g/kg of milk rather than the percentage of the total FA. Contrary to our results, precise equations (R^2^CV and/or R^2^V above 0.80), using MIR spectra and the g/kg of milk, were obtained to predict C4:0, C6:0, C8:0, C10:0, C12:0, C14:0, C16:0, cis9-C18:1, the sum of SFA, and the sum of MUFA [14,45,46]. Ferrand et al. [45] and Soyeurt et al. [14] reported a lower quality of prediction (R^2^CV and/or R^2^V below 0.75) for C18:2 n-6, C18:3 n-3, cis9trans11-C18:2, the sum of PUFA, n-3 FA, and n-6 FA than the FA previously described. Nevertheless, our predictions were lower than those of both studies. Contrary to many published studies [14,45,46], which generally use FOSS MilkoSan (transmission after homogenization) and fresh milk samples, in this study we used an ATR cell and frozen milk samples. Soyeurt et al. (2011) also added a bronopol preservative in their milk samples. This may partly explain the observed differences. The heterogenous structure after thawing can modify the MIR-ATR spectra and negatively modify the predictive quality for FA. However, according to the results of Aernouts et al. [19], the homogenization of milk samples does not allow one to improve predictions of fat, crude protein, lactose and urea using MIR-ATR. Further, Coppa et al. [12] did not observe any influence from thawing when predicting FA using MIR spectroscopy. The effect of thawing was overcome when we used NIR spectroscopy because we used the DESIR method [29]. According to our results, the NIR spectroscopy is adapted for predicting FA concentrations in frozen milk samples. This observation was confirmed by the relative uncertainty values calculated for the three spectroscopy methods for most FA. However, the use of the DESIR method prevents the application of this technology on-line during milking because this method requires dried milk samples.

## 5. Conclusions

This study is the first to compare the results obtained using three different spectroscopy techniques (NIR, MIR-ATR, and molecular fluorescence) to predict carotenoids, vitamins, and FA in the same frozen milk samples. Our hypothesis was that MIR and fluorescence techniques could be better adapted to predict milk compounds with low concentrations than NIR spectroscopy. Our results show that the NIR technique using the DESIR method is generally better adapted than both other techniques (MIR-ATR and fluorescence) to predict most carotenoids, vitamins and FA from frozen samples. However, we also determined that it is generally difficult to obtain high prediction quality when the milk compounds are of a low concentration, whatever the spectroscopy technique used. According to our encouraging results, it seems possible to obtain robust models to predict the milk concentrations of cis9-β-carotene, all-trans-β-carotene, β-cryptoxanthin, zeaxanthin, the sum of carotenoids, and vitamin A using NIR spectroscopy. Furthermore, a robust model could be obtained to predict the milk concentrations of lutein and α-tocopherol using MIR spectroscopy. Future studies should confirm and improve our prediction models. Indeed, the development of new prediction models would allow us to increase the number of milk compounds predicted simultaneously from the same spectrum. Consequently, it could be possible to consider to weigh the milk price, especially through a global quantitative evaluation of compounds of interest to human health. This approach could also be used to guarantee the fine composition of dairy products under sign of quality. Our new hypothesis is that it can be possible to improve the prediction quality of our models using fresh milk samples.

## Figures and Tables

**Table 1:** Summary of the source of all data, herds characteristics, type of samples and analysed compounds according to the experiments.

Experiment	Breeds	Mean Parity	Mean DIM (Days)	n	Type of Samples	Analysed Compounds
Exp. 1	Holstein (18.7%) Montbéliarde (80.3%)	2.6	182.0	62	Bulk milk	Carotenes
						Vitamins A and E
						Vitamins B_9_ and B_12_
						Fatty acids
Exp. 2	Holstein (100 %)	2.6	84.6	48	Individual milk	Carotenes
						Vitamins A and E
						Fatty acids
Exp. 3	Holstein (100 %)	2.7	130.2	108	Individual milk	Carotenes
						Vitamins A and E
						Fatty acids
Exp. 4	Holstein (100 %)	3.0	100.7	24	Individual milk	Carotenes
						Vitamins A and E

DIM: days-in-milk. Exp: experiment. n: number of dairy cows.

**Table 2:** Descriptive statistics of carotenoids, vitamins and selected fatty acids (FA, expressed in g/100 g of the total FA) in the calibration and validation sets used for near-, mid-infrared and fluorescence spectroscopy modelling.

Components	Calibration Set					SEL	Validation Set				
	*n*	Min	Max	Mean	SD		*n*	Min	Max	Mean	SD
Carotenoids											
cis13-β-carotene (µg/mL)	182	0.01	0.07	0.04	0.01	0.002	54	0.03	0.06	0.04	0.01
cis9-β-carotene (µg/mL)	182	<0.01	0.04	0.02	0.01	0.002	54	<0.01	0.04	0.02	0.01
All-trans-β-carotene (µg/mL)	182	0.05	0.58	0.23	0.11	0.030	54	0.07	0.58	0.24	0.10
Sum of β-carotenes (µg/mL)	182	0.10	0.62	0.29	0.11	-	54	0.11	0.63	0.30	0.11
β-cryptoxanthin (µg/mL)	182	<0.01	0.07	0.03	0.02	0.001	49	<0.01	0.06	0.03	0.02
Lutein (µg/mL)	177	0.03	0.37	0.17	0.07	0.010	51	0.03	0.28	0.17	0.07
Zeaxanthin (µg/mL)	181	<0.01	0.29	0.1	0.07	0.002	54	<0.01	0.22	0.10	0.07
Sum of Carotenoids (µg/mL)	182	0.11	1.04	0.58	0.21	-	54	0.20	0.97	0.60	0.22
Vitamins											
α-tocopherol (µg/mL)	184	<0.01	2.81	1.18	0.49	0.040	54	<0.01	2.79	1.32	0.59
γ-tocopherol (µg/mL)	165	0.39	1.28	0.67	0.18	-	52	0.39	1.11	0.70	0.18
Sum of tocopherols (µg/mL)	166	1.03	4.09	1.95	0.51	-	52	1.14	3.65	2.15	0.60
Vitamin A (µg retinol/mL)	184	0.03	1.33	0.52	0.24	0.040	49	0.03	0.97	0.48	0.20
Vitamin B_12_ (pg/mL)	48	1034.14	4826.39	2949.41	742.35	104.700	14	1923.32	4310.28	2903.59	666.13
Vitamin B_9_ (ng/mL)	48	82.86	137.78	108.13	12.16	3.130	14	94.38	152.66	110.75	14.47
FA (g/100g of the total FA)											
C4:0	166	1.59	3.74	2.68	0.40	0.110	52	1.48	3.76	2.66	0.41
C6:0	166	0.85	2.84	1.91	0.38	0.080	51	0.77	2.71	1.93	0.43
C8:0	166	0.29	1.85	1.17	0.31	0.060	47	0.21	1.69	1.18	0.35
C10:0	166	0.83	4.42	2.78	0.88	0.130	47	0.65	4.24	2.83	0.93
C12:0	166	1.08	5.81	3.38	1.06	0.130	47	0.93	4.98	3.41	1.11
C14:0	166	6.41	15.18	11.55	2.26	0.250	47	5.23	15.89	11.83	2.61
C16:0	166	14.15	40.36	28.07	5.96	0.190	47	16.72	38.28	28.77	5.79
C18:0	166	4.76	16.55	10.01	2.53	0.180	47	6.43	15.42	9.82	2.15
trans10-C18:1	166	0.03	1.50	0.39	0.26	-	47	0.09	1.46	0.39	0.27
trans11-C18:1	166	0.45	14.76	2.91	2.87	-	47	0.58	12.52	2.63	2.8
cis9-C18:1	166	11.32	31.1	19.36	4.24	0.030	47	12.58	29.55	19.30	4.48
trans11*cis*15-C18:2	166	0.01	4.17	0.30	0.50	-	46	<0.01	2.45	0.23	0.39
C18:2 n-6	166	0.68	2.17	1.21	0.29	0.030	52	0.6	1.51	1.15	0.22
C18:3 n-3	166	0.11	7.61	0.83	0.30	0.030	47	0.12	6.41	0.69	1.05
cis9trans11-C18:2	166	0.22	3.71	0.94	0.76	0.010	47	0.26	5.40	0.84	0.86
Sum of SFA	166	36.74	78.06	62.79	9.14	0.400	47	41.92	76.37	63.80	9.81
Sum of MUFA	166	17.73	50.65	30.80	8.48	0.460	47	18.44	49.15	30.26	9.03
Sum of PUFA	166	2.02	14.08	4.32	2.14	0.100	47	2.07	10.69	3.92	2.03
Sum of odd and/or branched FA	166	2.01	5.66	3.65	0.82	-	47	2.28	4.77	3.69	0.75
Sum of trans FA	166	0.35	29.05	6.85	6.92	0.200	52	0.33	27.17	6.55	6.93
n-3 FA	166	0.19	1.62	0.60	0.25	-	47	0.19	1.34	0.53	0.20
n-6 FA	166	0.84	2.42	1.45	0.29	-	51	0.93	1.74	1.40	0.21

*n*: number of samples. Min: minimum. Max: maximum. SD: standard deviation. SEL: standard error of laboratory were estimated according to the methodology proposed by AOAC [28]. The SEL of FA were taken from Coppa et al. [25]. -: not available. SFA: saturated FA. MUFA: monounsaturated FA. PUFA: polyunsaturated FA.

**Table 3:** Prediction of different carotenoids in cow milk according to the equations developed from three spectroscopy techniques: near-, mid-infrared (NIR and MIR, respectively) and fluorescence spectroscopy.

Carotenoids	Calibration Set									Validation Set		
	Spectral Processing	Spectroscopy Technique ^1^	n	Number of Outliers	T	SEC	R^2^C	SECV	R^2^CV	n	SEP	R^2^V
cis13-β-carotene (µg/mL)												
	None 1,4,4	NIR	179	3	3	0.01	0.34	0.01	0.27	54	0.01	0.14
	None 2,4,4	MIR-ATR	173	9	1	0.01	0.37	0.01	0.08		0.01	0.01
	SNV and Detrend 0,0,1	Fluorescence (lycopene)	162	12	2	0.01	0.38	0.01	0.33		0.01	0.09
cis9-β-carotene (µg/mL)												
	SNV and Detrend 1,4,4	NIR	174	8	4	0.01	0.74	0.01	0.72	54	0.01	0.61
	None 1,20,20	MIR-ATR	177	5	3	0.01	0.65	0.01	0.54		0.01	0.50
	None 0,0,1	Fluorescence (carotene)	167	12	8	0.01	0.86	0.01	0.47		0.01	0.24
All-trans-β-carotene (µg/mL)												
	None 0,0,1	NIR	177	5	9	0.05	0.70	0.06	0.61	54	0.08	0.32
	SNV and Detrend 1,30,30	MIR	175	7	2	0.07	0.40	0.08	0.31		0.10	0.11
	Weighted MSC 0,0,1	Fluorescence (carotene)	160	10	5	0.05	0.74	0.06	0.60		0.07	0.54
Sum of β-carotenes (µg/mL)												
	None 2,10,10	NIR	177	5	3	0.07	0.56	0.07	0.51	54	0.09	0.20
	Detrend 0,0,1	MIR-ATR	176	6	2	0.09	0.25	0.09	0.19		0.10	0.13
	SNV and Detrend 2,8,8	Fluorescence (carotene)	158	11	3	0.05	0.75	0.06	0.62		0.08	0.36
β-cryptoxanthin (µg/mL)												
	MSC 1,4,4	NIR	177	5	4	0.01	0.75	0.01	0.72	49	0.01	0.63
	Detrend 1,8,8	MIR-ATR	177	5	3	0.01	0.72	0.01	0.56		0.01	0.41
	Weighted MSC 0,0,1	Synchronous fluorescence	158	11	10	0.01	0.73	0.01	0.59		0.02	0.01
Lutein (µg/mL)												
	SNV and Detrend 1,4,4	NIR	172	5	3	0.04	0.67	0.04	0.64	51	0.06	0.09
	None 2,20,20	MIR-ATR	166	11	2	0.03	0.74	0.04	0.65		0.04	0.41
	SNV and Detrend 0,0,1	Fluorescence (carotene)	158	10	4	0.04	0.54	0.05	0.39		0.05	0.27
Zeaxanthin (µg/mL)												
	SNV and Detrend 1,4,4	NIR	174	7	4	0.03	0.80	0.03	0.77	54	0.04	0.67
	None 1,8,8	MIR-ATR	178	3	2	0.04	0.71	0.04	0.64		0.05	0.50
	None 0,0,1	Fluorescence (carotene)	168	10	5	0.05	0.52	0.05	0.45		0.05	0.42
Sum of Carotenoids (µg/mL)												
	MSC 1,4,4	NIR	181	1	4	0.12	0.66	0.13	0.60	54	0.15	0.50
	SNV and Detrend 1,30,30	MIR-ATR	171	11	2	0.13	0.53	0.14	0.48		0.19	0.20
	Standard MSC 0,0,1	Fluorescence (carotene)	165	11	4	0.13	0.50	0.15	0.38		0.17	0.30

ATR: attenuated total reflectance. n: number of samples. T: number of PLS terms in the model. SEC: standard error of calibration. R^2^C: coefficient of determination in calibration. SECV: standard error of cross-validation. SEP: standard error of prediction. R^2^V: coefficient of determination in validation. SNV: standard normal variate. MSC: multiplicative scatter correction. ^1^ Fluorescence molecules used to establish models are mentioned in parenthesis.

**Table 4:** Prediction of different vitamins in cow milk according to the equations developed from three spectroscopy techniques: near-, mid-infrared (NIR and MIR, respectively) and fluorescence spectroscopy.

Vitamins	Calibration Set									Validation Set		
	Spectral Processing	Spectroscopy Technique _1_	n	Number of Outliers	T	SEC	R_2_C	SECV	R_2_CV	n	SEP	R_2_V
α-tocopherol (µg/mL)												
	SNV and Detrend 0,0,1	NIR	176	8	3	0.30	0.54	0.30	0.52	54	0.52	0.01
	None 1,8,8	MIR-ATR	178	6	3	0.26	0.70	0.31	0.56		0.41	0.40
	Standard MSC 0,0,1	Fluorescence (carotene)	165	12	4	0.28	0.44	0.31	0.35		0.47	0.17
γ-tocopherol (µg/mL)												
	Detrend 1,4,4	NIR	161	4	1	0.16	0.09	0.16	0.07	52	0.17	0.06
	None 2,8,8	MIR-ATR	159	6	1	0.14	0.32	0.15	0.19		0.15	0.26
	SNV and Detrend 0,0,1	Fluorescence (lycopene)	158	7	2	0.14	0.23	0.15	0.16		0.18	0.05
Sum of tocopherols (µg/mL)												
	SNV and Detrend 2,10,10	NIR	162	4	1	0.46	0.10	0.47	0.05	52	0.57	0.08
	None 2,10,10	MIR-ATR	162	4	1	0.41	0.27	0.44	0.16		0.55	0.16
	Inverse MSC 0,0,1	Fluorescence (carotene)	159	7	3	0.38	0.32	0.44	0.15		0.60	0.03
Vitamin A (µg retinol/mL)												
	SNV and Detrend 1,4,4	NIR	174	10	3	0.12	0.69	0.13	0.65	49	0.15	0.34
	Detrend 0,0,1	MIR-ATR	178	6	5	0.15	0.58	0.17	0.46		0.16	0.27
	Standard MSC 0,0,1	Synchronous fluorescence	156	14	10	0.11	0.67	0.14	0.52		0.17	0.05
Vitamin B_12_ (pg/mL)												
	Detrend 0,0,1	NIR	48	0	1	724.1	0.05	772.98	0.01	14	611.82	0.16
	SNV and Detrend 1,20,20	MIR-ATR	43	5	1	604.27	0.15	673.92	0.01		907.3	0.18
	None 0,0,1	Synchronous fluorescence	38	2	1	707.26	0.19	776.66	0.05		918.32	0.05
Vitamin B_9_ (ng/mL)												
	MSC 0,0,1	NIR	48	0	3	10.58	0.24	12.05	0.03	14	37.95	0.69
	Weighted MSC 0,0,1	MIR-ATR	46	2	1	10.22	0.22	10.61	0.16		22.95	0.02
	Standard MSC 1,4,4	Fluorescence (carotene)	48	0	3	5.71	0.77	10.91	0.23		14.29	0.08

ATR: attenuated total reflectance. n: number of samples. T: number of PLS terms in the model. SEC: standard error of calibration. R^2^C: coefficient of determination in calibration. SECV: standard error of cross-validation. SEP: standard error of prediction. R^2^V: coefficient of determination in validation. SNV: standard normal variate. MSC: multiplicative scatter correction. ^1^ Fluorescence molecules used to establish models are mentioned in parenthesis.

**Table 5:** Prediction of different fatty acids (FA, expressed in g/100g of the total FA) in cow milk according to the equation developed from three spectroscopy techniques: near-, mid-infrared (NIR and MIR, respectively) and fluorescence spectroscopy.

FA	Calibration Set									Validation Set		
	Spectral Processing	Spectroscopy Technique ^1^	*n*	Number of Outliers	T	SEC	R^2^C	SECV	R^2^CV	n	SEP	R^2^V
C4:0												
	None 1,4,4	NIR	163	3	3	0.34	0.23	0.35	0.17	52	0.38	0.17
	None 0,0,1	MIR-ATR	161	5	5	0.32	0.30	0.35	0.15		0.42	0.04
	None 0,0,1	Fluorescence (lycopene)	163	3	1	0.39	0.01	0.40	0.01		0.41	0.01
C6:0												
	SNV and Detrend 2,10,10	NIR	161	5	5	0.18	0.76	0.20	0.72	51	0.17	0.85
	Detrend 1,4,4	MIR-ATR	164	2	1	0.32	0.22	0.39	0.01		0.44	0.01
	None 0,0,1	Fluorescence (carotene)	164	2	1	0.36	0.02	0.36	0.01		0.42	0.02
C8:0												
	SNV and Detrend 1,4,4	NIR	158	8	5	0.13	0.83	0.14	0.80	47	0.12	0.89
	SNV and Detrend 1,8,8	MIR-ATR	163	3	3	0.16	0.70	0.28	0.14		0.35	0.16
	Inverse MSC 2,8,8	Synchronous fluorescence	158	8	2	0.26	0.35	0.28	0.26		0.37	0.01
C10:0												
	SNV and Detrend 2,8,8	NIR	159	7	5	0.35	0.84	0.39	0.81	47	0.33	0.89
	None 1,8,8	MIR-ATR	163	3	3	0.46	0.72	0.81	0.15		0.91	0.18
	Inverse MSC 2,8,8	Synchronous fluorescence	158	8	2	0.71	0.40	0.78	0.28		0.97	0.01
C12:0												
	SNV and Detrend 1,4,4	NIR	160	6	6	0.43	0.84	0.49	0.79	47	0.37	0.90
	SNV 2,10,10	MIR-ATR	162	4	3	0.56	0.72	0.94	0.21		1.26	0.06
	Inverse MSC 2,8,8	Synchronous fluorescence	154	12	9	0.60	0.71	0.83	0.44		1.60	0.05
C14:0												
	SNV and Detrend 1,4,4	NIR	158	8	5	0.87	0.85	0.95	0.82	47	0.91	0.89
	SNV 1,8,8	MIR-ATR	165	1	4	1.07	0.78	2.14	0.12		2.61	0.19
	Detrend 1,4,4	Synchronous fluorescence	156	10	9	1.17	0.75	1.58	0.55		3.49	0.06
C16:0												
	SNV 1,4,4	NIR	157	9	7	2.08	0.87	2.55	0.81	47	2.38	0.85
	SNV and Detrend 1,8,8	MIR-ATR	166	0	3	3.42	0.66	5.73	0.06		6.25	0.14
	None 2,8,8	Synchronous fluorescence	155	11	4	3.95	0.56	4.24	0.49		7.29	0.03
C18:0												
	SNV and Detrend 1,4,4	NIR	164	2	10	1.40	0.69	1.70	0.55	47	1.48	0.60
	SNV and Detrend 1,8,8	MIR-ATR	160	6	3	1.24	0.71	2.12	0.17		2.64	0.08
	SNV and Detrend 2,8,8	Synchronous fluorescence	148	12	2	0.78	0.27	0.84	0.17		1.01	0.01
trans10-C18:1												
	SNV 1,4,4	NIR	154	10	4	0.10	0.46	0.11	0.38	47	0.22	0.35
	None 0,0,1	MIR-ATR	154	10	1	0.14	0.08	0.14	0.06		0.40	0.02
	SNV and Detrend 0,0,1	Fluorescence (vitamin A)	145	11	1	0.11	0.28	0.12	0.26		0.26	0.04
trans11-C18:1												
	SNV 1,4,4	NIR	154	10	10	0.68	0.91	0.83	0.87	47	1.39	0.79
	SNV and Detrend 2,30,30	MIR-ATR	153	11	1	1.49	0.17	1.59	0.06		2.81	0.09
	Detrend 1,4,4	Synchronous fluorescence	147	10	8	0.88	0.67	1.27	0.42		3.10	0.01
cis9-C18:1												
	MSC 1,4,4	NIR	161	5	9	1.69	0.83	2.26	0.70	47	2.08	0.79
	SNV 1,8,8	MIR-ATR	162	4	2	2.04	0.75	3.67	0.19		5.22	0.02
	Weighted MSC 1,4,4	Synchronous fluorescence	154	10	10	1.78	0.83	3.01	0.51		6.74	0.12
trans11*cis*15-C18:2												
	None 2,10,10	NIR	158	8	4	0.12	0.55	0.13	0.50	46	0.35	0.34
	SNV and Detrend 1,8,8	MIR-ATR	156	10	3	0.10	0.68	0.17	0.18		0.39	0.10
	Inverse MSC 0,0,1	Fluorescence (fluorescent oxidation products)	144	12	4	0.13	0.31	0.14	0.20		0.69	0.01
C18:2 n-6												
	SNV and Detrend 2,10,10	NIR	159	7	2	0.18	0.53	0.19	0.47	52	0.18	0.49
	None 2,30,30	MIR-ATR	163	3	2	0.21	0.42	0.23	0.32		0.19	0.34
	None 0,0,1	Fluorescence (carotene)	156	10	4	0.17	0.56	0.18	0.50		0.16	0.50
C18:3 n-3												
	Detrend 1,4,4	NIR	156	10	4	0.24	0.39	0.28	0.16	47	1.07	0.07
	SNV and Detend 1,8,8	MIR	163	3	1	0.17	0.23	0.19	0.01		0.13	0.28
	Detrend 1,1,4	Synchronous fluorescence	150	10	8	0.09	0.76	0.12	0.56		0.19	0.09
cis9trans11-C18:2												
	None 2,10,10	NIR	159	7	3	0.46	0.46	0.48	0.39	47	0.67	0.47
	SNV 2,30,30	MIR	155	11	1	0.64	0.19	0.69	0.09		1.23	0.10
	Detrend 1,1,4	Synchronous fluorescence	146	12	9	0.33	0.76	0.55	0.41		1.40	0.02
Sum of SFA												
	SNV and Detrend 1,4,4	NIR	160	6	8	2.37	0.69	2.53	0.65	47	2.03	0.96
	Standard MSC 1,4,4	MIR-ATR	161	5	2	3.79	0.80	7.85	0.16		12.63	0.03
	None 0,0,1	Synchronous fluorescence	161	5	1	8.89	0.07	8.89	0.07		13.76	0.01
Sum of MUFA												
	MSC 1,4,4	NIR	161	5	7	3.31	0.85	4.01	0.78	47	4.13	0.81
	SNV and Detrend 1,8,8	MIR-ATR	163	3	3	3.80	0.71	6.49	0.16		7.94	0.15
	Weighted MSC 2,8,8	Synchronous fluorescence	153	13	9	3.49	0.78	4.89	0.57		11.3	0.08
Sum of PUFA												
	MSC 2,10,10	NIR	157	9	9	0.62	0.85	0.76	0.78	47	0.95	0.80
	SNV 2,30,30	MIR-ATR	155	11	1	1.11	0.21	1.23	0.05		2.01	0.13
	Detrend 1,4,4	Synchronous fluorescence	149	12	2	1.08	0.19	1.25	0.20		2.39	0.01
Sum of odd and/or branched FA												
	SNV and Detrend 1,4,4	NIR	163	3	9	0.41	0.74	0.51	0.60	47	0.61	0.48
	SNV 1,30,30	MIR-ATR	158	8	8	0.35	0.82	0.55	0.53		0.78	0.19
	Inverse MSC 2,8,8	Synchronous fluorescence	155	11	5	0.45	0.65	0.51	0.57		0.77	0.03
Sum of trans FA												
	SNV and Detrend 1,4,4	NIR	157	9	10	1.59	0.94	2.31	0.88	52	2.95	0.84
	None 2,16,16	MIR-ATR	155	11	2	3.33	0.56	3.88	0.40		5.82	0.30
	None 0,0,1	Fluorescence (carotene)	154	12	5	3.27	0.58	3.97	0.44		5.52	0.37
n-3 FA												
	SNV and Detrend 2,10,10	NIR	162	4	9	0.16	0.52	0.20	0.26	47	0.19	0.29
	SNV and Detrend 1,20,20	MIR-ATR	162	4	1	0.20	0.19	0.22	0.04		0.20	0.11
	Standard MSC 1,4,4	Synchronous fluorescence	153	11	5	0.13	0.59	0.16	0.39		0.15	0.47
n-6 FA												
	MSC 2,10,10	NIR	162	4	2	0.21	0.34	0.23	0.28	51	0.19	0.30
	SNV 0,0,1	MIR-ATR	162	4	7	0.19	0.49	0.24	0.27		0.32	0.03
	None 0,0,1	Fluorescence (carotene)	160	6	4	0.20	0.43	0.22	0.33		0.20	0.21

ATR: attenuated total reflectance. n: number of samples. T: number of PLS terms in the model. SEC: standard error of calibration. R^2^C: coefficient of determination in calibration. SECV: standard error of cross-validation. SEP: standard error of prediction. R^2^V: coefficient of determination in validation. SFA: saturated FA. PUFA: polyunsaturated FA. SNV: standard normal variate. MSC: multiplicative scatter correction. ^1^ Fluorescence molecules used to establish models are mentioned in parenthesis.

**Table 6:** Bias and standard error of prediction corrected for bias (SEPc) for milk carotenoids, vitamins and selected fatty acids (FA, expressed in g 100/g of the total FA) obtained when each sample of validation set was predicted using calibration equation selected according to the spectroscopy technique: mid-infrared using an Attenuated Total Reflectance cell (MIR-ATR), near-infrared (NIR) and molecular fluorescence.

Components	Bias			SPEc		
	MIR-ATR	NIR	Fluorescence	MIR-ATR	NIR	Fluorescence
Carotenoids						
cis13-β-carotene (µg/mL)	0.002	0.002	0.005 *	0.01	0.009	0.009
cis*9*-β-carotene (µg/mL)	0.009	0.001	0.01 *	0.009 ^ab^	0.008 ^a^	0.01 ^b^
All-trans-β-carotene (µg/mL)	0.01	0.02 *	0.03 *	0.10 ^a^	0.08 ^ab^	0.07 ^b^
Sum of β-carotenes (µg/mL)	0.01	0.01	0.03 *	0.10	0.09	0.08
β-cryptoxanthin (µg/mL)	0.002	0.003 *	0.01 *	0.01 ^a^	0.01 ^a^	0.02 ^b^
Lutein (µg/mL)	0.01	0.01	0.03 *	0.04 ^a^	0.06 ^b^	0.05 ^ab^
Zeaxanthin (µg/mL)	0.004	0.005	0.02 *	0.05	0.04	0.05
Sum of Carotenoids (µg/mL)	0.02	0.01	0.09 *	0.19 ^a^	0.15 ^b^	0.17 ^ab^
Vitamins						
α-tocopherol (µg/mL)	0.17 *	0.17 *	0.21 *	0.41 ^a^	0.52 ^b^	0.47 ^ab^
γ-tocopherol (µg/mL)	0.06 *	0.05 *	0.01	0.15	0.17	0.18
Sum of tocopherols (µg/mL)	0.24 *	0.25 *	0.28 *	0.55	0.57	0.60
Vitamin A (µg retinol/mL)	−0.03	−0.02	0.09 *	0.16	0.15	0.17
Vitamin B_12_ (pg/mL)	212.83	−67.08	290.98	907.3	611.82	918.32
Vitamin B_9_ (ng/mL)	−4.85	8.81	2.16	22.95 ^ab^	37.95 ^a^	14.29 ^b^
FA (g 100/g of the total FA)						
C4:0	−0.04	−0.02	−0.03	0.42	0.38	0.41
C6:0	−0.04	−0.002	0.0002	0.44 ^a^	0.17 ^b^	0.42 ^a^
C8:0	−0.03	−0.01	0.10	0.35 ^a^	0.12 ^b^	0.37 ^a^
C10:0	−0.07	0.04	0.25	0.91 ^a^	0.33 ^b^	0.97 ^a^
C12:0	−0.13	−0.04	0.23	1.26 ^a^	0.37 ^b^	1.60 ^a^
C14:0	−0.06	0.05	0.05	2.61 ^a^	0.91 ^b^	3.49 ^a^
C16:0	−0.33	0.42	1.70	6.25 ^a^	2.38 ^b^	7.29 ^a^
C18:0	0.04	−0.39	0.13	2.64 ^a^	1.48 ^b^	1.01 ^c^
trans10-C18:1	0.03	0.08 *	0.02	0.42 ^a^	0.23 ^b^	0.26 ^b^
trans11-C18:1	0.40	−0.10	0.57	2.81 ^a^	1.39 ^b^	3.10 ^a^
cis9-C18:1	1.02	0.006	−0.36	5.22 ^a^	2.08 ^b^	6.74 ^a^
trans11cis15-C18:2	0.04	0.03	0.15	0.39 ^a^	0.35 ^a^	0.69 ^b^
C18:2 n-6	−0.05 *	−0.06 *	−0.10 *	0.19	0.18	0.16
C18:3 n-3	−0.06 *	0.2	−0.009	0.13 ^a^	1.07 ^b^	0.19 ^c^
cis9trans11-C18:2	0.17	−0.02	0.19	1.23 ^a^	0.67 ^b^	1.40 ^a^
Sum of SFA	−3.12	−0.07	−26.91 *	12.63 ^a^	2.03 ^b^	13.76 ^a^
Sum of MUFA	0.89	−0.69	−3.58 *	7.94 ^a^	4.13 ^b^	11.30 ^c^
Sum of PUFA	0.10	−0.14	0.36	2.01 ^a^	0.95 ^b^	2.39 ^a^
Sum of odd and/or branched FA	−0.05	0.05	−0.08	0.78	0.61	0.77
Sum of trans FA	1.03	−0.15	1.14	5.82 ^a^	2.95 ^b^	5.52 ^a^
n-3 FA	−0.04	−0.03	−0.04	0.20 ^a^	0.19 ^ab^	0.15 ^b^
n-6 FA	−0.05	−0.04	−0.08 *	0.32 ^a^	0.19 ^b^	0.20 ^b^

SFA: saturated FA. MUFA: monounsaturated FA. PUFA: polyunsaturated FA. Values on the same line, which had different letter (a-c) were significantly different (*p* ≤ 0.05). * Values of bias were significantly different from zero (*p* ≤ 0.05).

## Appendix A

### Description of the four Experimental Objectives

The objective of experiment 1 (*n* = 62) was to determine the effect of the production conditions on milk’s micronutrient composition. In this experiment, the bulk milk samples came from 32 commercial farms in three French regions (Normandie, Mont-du-Vivarais, and Isère) and were collected twice during the year (spring and winter). The farms were selected to cover a wide variability of dairy production conditions, particularly forage systems (grazed or preserved forage from permanent diversified or less diversified pastures or corn silage). For our study, only 62 samples were usable because, for others, there was not enough milk to perform the analyses by the three spectroscopy methods.

Individual milk samples came from three feeding experiments (experiments 2, 3 and 4) performed at the institut national de la recherche pour l’agriculture, l’alimentation et l’environnement (INRAE, UE Herbipole, Marcenat, France). The objective of experiments 2 and 3 was to analyse the effect of lipid supplementation on FA and antioxidant compound concentrations in milk (*n* = 156) (Agilait project: Structures, oxidation stability, properties and bioaccessibility of the milk fat of dairy products enriched in unsaturated fatty acids, funded by the Programme National de Recherche en Alimentation, Project No. ANR-06-PNRA-012). Experiments 2 and 3 were subdivided into two periods: pre-experimental and experimental. During the pre-experimental period of experiment 2, 24 cows received a diet based on grass and corn silages and dehydrated alfalfa. Then, during the experimental period, these cows were divided into four groups and received the same basal diet supplemented (5% of dry matter (DM)) or not with linseed oil, sunflower oil, or oleic sunflower oil, respectively. One milk sample was collected per cow at the end of both the pre-experimental and experimental periods. During the pre-experimental period of experiment 3, 36 cows received a diet based on grass and corn silages (30/70). Then, during the experimental period, the 36 cows were divided into six groups where three groups received a grass silage-based diet supplemented or not with linseed oil or rapeseed oil (5% of DM); the three other groups received corn silage-based diets supplemented or not with linseed oil or rapeseed oil (5% of DM). For each cow, one milk sample was collected during the pre-experimental period and two during the experimental period.

The objective of experiment 4 (*n* = 24), using 12 cows, was to analyse the effects of olive mill wastewater (OMWW) supplementation on milk composition [47]. This experiment took place at the pilot farm of Kadri Brahim (Université Mentouri, Constantine, Algeria). During the pre-experimental period, the cows received, on a DM basis 8 kg of forage (42% vetch-oat silage, 32% vetch-oat hay and 25% alfalfa hay) and 4.3 kg of concentrate (50% corn, 28% bran wheat and 22% soybean). During the experimental period, the cows received the same diet supplemented or not with OMWW (0.12 kg DM/day). One milk sample was collected per cow per period.

## Appendix C

**Table A1:** Descriptive statistics of selected fatty acids (FA, expressed in g fat/kg of milk) in the calibration and validation sets used for near-infrared, mid-infrared and fluorescence spectroscopy modelling.

Components	Calibration Set					Validation Set				
	*n*	Min	Max	Mean	SD	n	Min	Max	Mean	SD
FA										
C4:0	166	0.44	1.62	0.99	0.19	52	0.41	1.56	0.99	0.21
C6:0	166	0.22	1.10	0.71	0.18	51	0.21	1.13	0.72	0.23
C8:0	166	0.11	0.70	0.44	0.14	47	0.07	0.74	0.45	0.16
C10:0	166	0.26	1.86	1.04	0.38	47	0.20	1.77	1.08	0.42
C12:0	166	0.39	2.35	1.27	0.46	47	0.29	2.23	1.30	0.51
C14:0	166	1.86	6.29	4.31	1.11	47	1.64	6.60	4.46	1.28
C16:0	166	4.54	18.39	10.54	3.00	47	5.06	17.15	10.86	3.08
C18:0	166	1.59	6.22	3.69	0.98	47	2.24	6.47	3.65	0.92
trans10-C18:1	166	0.01	0.46	0.14	0.08	47	0.04	0.42	0.14	0.08
trans11-C18:1	166	0.19	3.83	0.99	0.81	47	0.26	4.94	0.92	0.91
cis9-C18:1	166	3.92	11.31	7.09	1.54	47	4.83	11.27	7.10	1.48
trans11cis15-C18:2	166	<0.01	1.20	0.10	0.15	46	<0.01	0.97	0.08	0.14
C18:2 n-6	166	0.21	0.87	0.45	0.13	52	0.18	0.66	0.43	0.11
C18:3 n-3	166	0.04	0.50	0.17	0.08	47	0.04	0.23	0.14	0.05
cis9trans11-C18:2	166	0.09	1.94	0.44	0.38	47	0.09	1.78	0.40	0.37
Sum of SFA	166	9.78	33.67	23.07	5.15	47	11.71	35.12	23.60	5.77
Sum of MUFA	166	5.94	16.33	10.61	2.22	47	6.83	16.01	10.52	2.36
Sum of PUFA	166	0.80	4.01	1.54	0.60	47	0.70	4.09	1.42	0.63
Sum of odd and/or branched FA	166	0.66	2.22	1.36	0.39	47	0.68	2.29	1.38	0.37
Sum of trans FA	166	0.60	8.57	2.74	1.84	52	0.86	10.72	2.60	1.98
n-3 FA	166	0.07	0.60	0.22	0.10	47	0.08	0.42	0.19	0.07
n-6 FA	166	0.30	0.97	0.54	0.13	51	0.31	0.80	0.52	0.11

*n*: number of samples. Min: minimum. Max: maximum. SD: standard deviation. FA: fatty acids. SFA: saturated FA. MUFA: monounsaturated FA. PUFA: polyunsaturated FA.

**Table A2:** Prediction of different fatty acids (FA, expressed in g fat/kg of milk) in cow milk according to the equation developed from three spectroscopy techniques: near-, mid-infrared (NIR and MIR, respectively) and fluorescence spectroscopy.

FA	Calibration Set									Validation Set		
	Spectral Processing	Spectroscopy Technique ^1^	*n*	Number of Outliers	T	SEC	R^2^C	SECV	R^2^CV	n	SEP	R^2^V
C4:0												
	SNV and Detrend 1,4,4	NIR	162	4	3	0.14	0.39	0.14	0.35	52	0.17	0.34
	SNV and Detrend 1,8,8	MIR-ATR	162	4	2	0.13	0.41	0.18	0.01		0.22	0.005
	None 0,0,1	Fluorescence (lycopene)	160	6	1	0.17	0.01	0.17	0.01		0.21	0.01
C6:0												
	SNV and Detrend 1,4,4	NIR	161	5	7	0.08	0.80	0.09	0.74	51	0.09	0.80
	SNV and Detrend 1,8,8	MIR-ATR	159	7	1	0.15	0.21	0.17	0.06		0.20	0.06
	None 0,0,1	Fluorescence (carotene)	164	2	1	0.18	0.03	0.18	0.01		0.20	0.08
C8:0												
	SNV and Detrend 1,4,4	NIR	156	10	7	0.05	0.87	0.06	0.82	47	0.06	0.86
	SNV and Detrend 1,8,8	MIR-ATR	163	3	3	0.07	0.70	0.13	0.14		0.15	0.18
	None 2,8,8	Synchronous fluorescence	147	13	6	0.09	0.63	0.10	0.53		0.11	0.52
C10:0												
	SNV and Detrend 1,4,4	NIR	160	6	6	0.15	0.84	0.17	0.79	47	0.17	0.85
	SNV and Detrend 1,8,8	MIR-ATR	163	3	4	0.18	0.76	0.34	0.19		0.44	0.11
	Inverse MSC 2,8,8	Synchronous fluorescence	147	12	5	0.24	0.60	0.27	0.49		0.32	0.42
C12:0												
	SNV and Detrend 1,4,4	NIR	161	5	5	0.19	0.83	0.22	0.79	47	0.22	0.82
	SNV and Detrend 1,8,8	MIR-ATR	162	4	4	0.22	0.75	0.4	0.21		0.54	0.09
	Inverse MSC 2,8,8	Synchronous fluorescence	148	11	5	0.29	0.60	0.34	0.47		0.37	0.46
C14:0												
	SNV and Detrend 1,4,4	NIR	159	7	6	0.41	0.87	0.44	0.84	47	0.50	0.85
	SNV and Detrend 1,8,8	MIR-ATR	166	0	3	0.62	0.68	1.07	0.06		1.20	0.20
	Detrend 1,4,4	Synchronous fluorescence	149	10	7	0.64	0.69	0.79	0.53		0.91	0.49
C16:0												
	SNV and Detrend 1,4,4	NIR	159	7	4	1.27	0.81	1.38	0.78	47	1.43	0.80
	SNV and Detrend 1,8,8	MIR-ATR	165	1	3	1.72	0.67	2.85	0.09		3.05	0.16
	SNV 2,8,8	Synchronous fluorescence	149	11	6	1.79	0.66	2.16	0.51		2.34	0.49
C18:0												
	SNV and Detrend 1,4,4	NIR	164	2	9	0.58	0.64	0.74	0.44	47	0.79	0.27
	SNV and Detrend 1,8,8	MIR-ATR	164	2	3	0.49	0.75	0.93	0.11		1.10	0.05
	SNV and Detrend 2,8,8	Synchronous fluorescence	155	11	3	0.75	0.37	0.83	0.24		0.94	0.04
trans10-C18:1											
	SNV and Detrend 1,4,4	NIR	157	9	3	0.04	0.37	0.04	0.32	47	0.06	0.38
	SNV and Detrend 1,8,8	MIR-ATR	154	12	3	0.02	0.71	0.05	0.01		0.09	0.02
	SNV and Detrend 0,0,1	Fluorescence (vitamin A)	131	15	4	0.03	0.36	0.04	0.28		0.23	0.01
trans11-C18:1											
	SNV and Detrend 1,4,4	NIR	155	11	10	0.20	0.92	0.24	0.88	47	0.50	0.73
	SNV and Detrend 1,8,8	MIR-ATR	156	10	3	0.27	0.70	0.54	0.17		0.87	0.17
	Detrend 1,4,4	Synchronous fluorescence	141	13	8	0.34	0.66	0.48	0.47		1.36	0.16
cis9-C18:1												
	SNV and Detrend 1,4,4	NIR	159	7	10	0.70	0.79	0.93	0.63	47	0.98	0.55
	SNV and Detrend 1,8,8	MIR-ATR	157	9	1	0.18	0.21	1.37	0.02		1.44	0.05
	Weighted MSC 1,4,4	Synchronous fluorescence	150	13	3	1.03	0.50	1.17	0.35		1.70	0.06
trans11cis15-C18:2												
	SNV and Detrend 1,4,4	NIR	157	9	3	0.05	0.43	0.05	0.41	46	0.14	0.19
	SNV and Detrend 1,8,8	MIR-ATR	152	14	2	0.04	0.48	0.06	0.13		0.14	0.13
	Inverse MSC 0,0,1	Fluorescence (fluorescent oxidation products)	145	13	1	0.05	0.13	0.06	0.13		0.16	0.01
C18:2 n-6												
	SNV and Detrend 1,4,4	NIR	159	7	9	0.06	0.72	0.08	0.53	52	0.09	0.45
	SNV and Detrend 1,8,8	MIR-ATR	162	4	3	0.07	0.70	0.10	0.37		0.09	0.36
	None 0,0,1	Fluorescence using carotene	161	5	5	0.07	0.60	0.08	0.49		0.09	0.44
C18:3 n-3												
	SNV and Detrend 1,4,4	NIR	164	2	7	0.06	0.43	0.07	0.25	47	0.05	0.26
	SNV and Detrend 1,8,8	MIR-ATR	162	4	1	0.06	0.23	0.07	0.06		0.05	0.23
	Detrend 1,4,4	Synchronous fluorescence	143	14	8	0.03	0.74	0.04	0.61		0.11	0.10
cis9trans11-C18:2												
	SNV and Detrend 1,4,4	NIR	149	17	8	0.10	0.85	0.13	0.76	47	0.21	0.71
	SNV and Detrend 1,8,8	MIR-ATR	155	11	3	0.12	0.73	0.22	0.18		0.35	0.21
	Detrend 1,4,4	Synchronous fluorescence	137	15	8	0.14	0.68	0.19	0.50		0.32	0.33
Sum of SFA												
	SNV and Detrend 1,4,4	NIR	161	5	5	2.08	0.83	2.25	0.80	47	2.79	0.78
	SNV and Detrend 1,8,8	MIR-ATR	165	1	3	2.86	0.69	4.96	0.06		5.39	0.15
	None 0,0,1	Synchronous fluorescence	148	12	8	3.38	0.58	3.84	0.48		4.15	0.50
Sum of MUFA												
	SNV and Detrend 1,4,4	NIR	158	8	8	0.95	0.80	1.20	0.67	47	1.18	0.74
	SNV and Detrend 1,8,8	MIR-ATR	164	2	3	1.2	0.69	1.97	0.18		2.4	0.10
	Weighted MSC 2,8,8	Synchronous fluorescence	149	10	5	1.42	0.56	1.60	0.45		2.50	0.11
Sum of PUFA												
	SNV and Detrend 1,4,4	NIR	156	10	8	0.21	0.78	0.26	0.66	47	0.33	0.75
	SNV and Detrend 1,8,8	MIR-ATR	156	10	4	0.21	0.76	0.36	0.22		0.66	0.07
	Detrend 1,4,4	Synchronous fluorescence	141	11	8	0.24	0.63	0.34	0.33		0.60	0.21
Sum of odd and/or branched FA												
	SNV and Detrend 1,4,4	NIR	163	3	9	0.16	0.82	0.19	0.75	47	0.25	0.54
	SNV and Detrend 1,8,8	MIR-ATR	165	1	2	0.28	0.48	0.34	0.23		0.29	0.29
	Inverse MSC 2,8,8	Synchronous fluorescence	149	13	6	0.19	0.72	0.21	0.65		0.71	0.06
Sum of trans FA												
	SNV and Detrend 1,4,4	NIR	155	11	10	0.38	0.95	0.49	0.92	52	0.9	0.82
	SNV and Detrend 1,8,8	MIR-ATR	155	11	1	1.19	0.23	1.30	0.09		1.89	0.09
	None 0,0,1	Fluorescence (carotene)	153	13	2	1.13	0.23	1.21	0.22		1.89	0.07
n-3 FA												
	SNV and Detrend 1,4,4	NIR	161	5	1	0.08	0.18	0.08	0.15	47	0.07	0.01
	SNV and Detrend 1,8,8	MIR-ATR	162	4	1	0.08	0.26	0.09	0.04		0.12	0.15
	Standard MSC 1,4,4	Synchronous fluorescence	143	11	4	0.05	0.63	0.06	0.48		0.06	0.31
n-6 FA												
	SNV and Detrend 1,4,4	NIR	160	6	1	0.09	0.49	0.10	0.42	51	0.09	0.44
	SNV and Detrend 1,8,8	MIR-ATR	160	6	2	0.09	0.48	0.11	0.31		0.07	0.10
	None 0,0,1	Fluorescence (carotene)	162	4	6	0.08	0.61	0.10	0.34		0.10	0.33

ATR: attenuated total reflectance. n: number of samples. T: number of PLS terms in the model. SEC: standard error of calibration. R^2^C: coefficient of determination in calibration. SECV: standard error of cross-validation. SEP: standard error of prediction. R^2^V: coefficient of determination in validation. SFA: saturated FA. MUFA: monounsaturated FA. PUFA: polyunsaturated FA. SNV: standard normal variate. ^1^ Fluorescence molecules used to establish models are mentioned in parenthesis.

**Table A3:** Bias and standard error of prediction corrected for bias (SEPc) for selected milk fatty acids (FA, expressed in g fat/kg of milk) obtained when each sample of validation set was predicted using calibration equation selected according to the spectroscopy technique: mid-infrared using an Attenuated Total Reflectance cell (MIR-ATR), near-infrared (NIR) and fluorescence.

Components	Bias			SPEc		
	MIR-ATR	NIR	Fluorescence	MIR-ATR	NIR	Fluorescence
FA						
C4:0	0.0005	−0.003	0.03	0.22 ^a^	0.17 ^b^	0.21 ^a^
C6:0	0.004	−0.003	0.04	0.20 ^a^	0.09 ^b^	0.20 ^a^
C8:0	0.0003	−0.005	0.04 *	0.15 ^a^	0.06 ^b^	0.11 ^c^
C10:0	0.01	−0.01	0.07	0.44 ^a^	0.17 ^b^	0.32 ^c^
C12:0	0.004	−0.04	0.08	0.54 ^a^	0.22 ^b^	0.37 ^c^
C14:0	0.03	0.02	0.55 *	1.20 ^a^	0.50 ^b^	0.91 ^a^
C16:0	−0.07	0.04	0.54	3.05 ^a^	1.43 ^b^	2.34 ^a^
C18:0	−0.12	−0.23	0.07	1.10 ^a^	0.79 ^b^	0.94 ^ab^
trans10-C18:1	0.02	0.02 *	0.03	0.09 ^a^	0.06 ^b^	0.23 ^c^
trans11-C18:1	0.11	−0.008	0.42 *	0.87 ^a^	0.50 ^b^	1.36 ^c^
cis9-C18:1	−0.002	−0.04	0.02	1.44 ^a^	0.98 ^b^	1.70 ^a^
trans11cis15-C18:2	0.01	0.01	0.02	0.14	0.14	0.16
C18:2 n-6	−0.02	−0.01	−0.05	0.09	0.09	0.09
C18:3 n-3	−0.02 *	−0.02 *	0.003	0.05 ^a^	0.05 ^a^	0.11 ^b^
cis9trans11-C18:2	0.04	0.01	0.11 *	0.35 ^a^	0.21 ^b^	0.32 ^a^
Sum of SFA	0.10	−0.26	0.58	5.39 ^a^	2.79 ^b^	4.15 ^a^
Sum of MUFA	−0.09	−0.08	−0.11	2.40 ^a^	1.18 ^b^	2.50 ^a^
Sum of PUFA	−0.01	−0.005	0.06	0.66 ^a^	0.33 ^b^	0.60 ^a^
Sum of odd and/or branched FA	−0.01	0.01	0.04	0.29 ^a^	0.25 ^a^	0.71 ^b^
Sum of trans FA	0.27	−0.01	0.12	1.89 ^a^	0.90 ^b^	1.89 ^a^
n-3 FA	−0.02 *	−0.03 *	−0.002	0.07	0.07	0.06
n-6 FA	−0.02	−0.01	−0.02	0.12	0.09	0.10

SFA: saturated FA. MUFA: monounsaturated FA. PUFA: polyunsaturated FA. Values on the same line, which had different letter (a-c) were significantly different (*p* ≤ 0.05). * Values of bias were significantly different from zero (*p* ≤ 0.05).

**Table A4:** Correlation matrix (Pearson’s correlations) between milk compounds when |*r*| ≥ 0.80, obtained on the calibration set.

	C6:0	C8:0	C10:0	C12:0	C14:0	C16:0	trans11-C18:1	cis9-C18:1	C18:2 n-6	C18:3 n-3	cis9trans11-C18:2	Sum of SFA	cis13-ßcarotene	cis9-ß-carotene	ß-cryptoxanthin	Lutein	α-tocopherol	γ-tocopherol
C8:0	0.95																	
C10:0	0.90	0.97																
C12:0	0.86	0.94	0.99															
C14:0	0.86	0.93	0.95	0.96														
C16:0		0.80	0.81	0.83	0.86													
cis9-C18:1			−0.83	−0.84	−0.83	−0.81												
cis9trans11-C18:2							0.97											
Sum of SFA	0.88	0.90	0.92	0.91	0.93	0.94	−0.84	−0.80			−0.83							
Sum of MUFA	−0.88	−0.91	−0.93	−0.92	−0.94	−0.94		0.87				−0.98						
Sum of PUFA							0.91				0.90	−0.85						
n-3 FA										0.95								
n-6 FA									0.90									
ß-cryptoxanthin														0.89				
Lutein														0.84	0.79			
Zeaxanthin														0.95	0.87	0.87		
Sum of carotenoids													0.81		0.84	0.85		
Sum of tocopherols																	0.97	0.91

In this correlation matrix, the fatty acids (FA) are expressed in g 100/g of the total FA and the carotenoids and vitamins are expressed in µg/mL.
